# Thermal behavior of spin-current generation in Pt_*x*_Cu_1-*x*_ devices characterized through spin-torque ferromagnetic resonance

**DOI:** 10.1038/s41598-020-66762-8

**Published:** 2020-06-15

**Authors:** G. D. H. Wong, W. C. Law, F. N. Tan, W. L. Gan, C. C. I. Ang, Z. Xu, C. S. Seet, W. S. Lew

**Affiliations:** 1 School of Physical & Mathematical Sciences, Nanyang Technological University, 21 Nanyang Link, Singapore, 637371 Singapore; 2 GLOBALFOUNDRIES Singapore Pte, Ltd., Singapore, 738406 Singapore

**Keywords:** Condensed-matter physics, Condensed-matter physics

## Abstract

High temperature studies of spin Hall effect have often been neglected despite its profound significance in real-world devices. In this work, high temperature spin torque ferromagnetic resonance measurement was performed to evaluate the effects of temperature on the Gilbert damping and spin Hall efficiency of Pt_*x*_Cu_1−*x*_. When the temperature was varied from 300 K to 407 K, the Gilbert damping was relatively stable with a change of 4% at composition *x* = 66%. Alloying Pt and Cu improved the spin Hall efficiency of Pt_75_Cu_25_/Co/Ta by 29% to a value of 0.31 ± 0.03 at 407 K. However, the critical switching current density is dependent on the ratio between the Gilbert damping and spin Hall efficiency and the smallest value was observed when *x* = 47%. It was found that at this concentration, the spin transparency was at its highest at 0.85 ± 0.09 hence indicating the importance of interfacial transparency for energy efficient devices at elevated temperature.

## Introduction

Current induced spin-orbit torque (SOT) has attracted a remarkable amount of attention in the field of spintronics due to its ability to manipulate magnetization^1–6^. SOT based magnetic memory serves as an alternative for energy efficient memory as compared to spin transfer torque magnetic random-access memory (STT-MRAM)^7^. In a heavy-metal/ferromagnetic layer (HM/FM) system, the SOT is contributed by two well-known phenomena; the spin Hall effect (SHE) in HM^3,6,8–11^ and/or the Rashba-Edelstein effect at their interface^12–14^. In the former, a transverse spin current ${J}_{S}$ is generated from charge current ${J}_{C}$ passing through material of strong spin-orbit interaction and injected into the adjacent FM to exert an STT on the magnetization. The magnetization switching efficiency depends on the charge-to-spin current conversion ratio which is defined as the spin Hall angle ${\theta }_{SH}=(2e/\hslash ){J}_{S}/{J}_{C}$^1,4^.

Alloying is an established approach to enhance spin Hall efficiency by increasing the extrinsic contribution of SHE and this has been shown in many previous studies. *R. Ramaswamy* reported that with as little as *x* = 28% in Pt_*x*_Cu_1−*x*_, one can achieve similar spin Hall efficiency as pure Pt. From his study, an enhancement in spin Hall efficiency was observed at room temperature^15^. However, it is also important to characterize the SHE at the elevated temperatures that MRAM devices are often operated at. During MRAM switching, a large current is required resulting in temperature rise that could potentially lead to detrimental effects on the device performance. Temperature studies using various characterization techniques including second harmonic measurement^16–19^, spin-pumping^20^ and spin-transfer ferromagnetic resonance (ST-FMR)^21^ have been explored but only within and below room temperature^22^. Although the effects of elevated temperature on SHE is crucial for device applications^23,24^, the quest for high temperature SHE characterization remains relatively uncharted. In a recent ST-FMR study by *P. Phu*, a joule heating technique was applied to momentarily raise the temperature of the sample during characterization^22^. The usage of a heating element as deployed in our experimental setup could provide consistent heating similar to external stimulus for investigating the thermal dependence of spin conductance measurements.

In this work, we demonstrated that alloying can help to improve the thermal robustness of spin generation in high spin orbit coupling (SOC) materials. This was evident by performing *in-situ* high temperature ST-FMR measurement on our sample, Pt_*x*_Cu_1−*x*_/Co/Ta. The results revealed that both the spin Hall efficiency and the thermal stability are enhanced in Pt_*x*_Cu_1−*x*_ alloys. The highest spin transparency was observed in the alloy with the smallest switching current density. Our work indicates that there is a relation between the three components of a spin torque generator - switching current density, diffusion length and spin transparency. This work provides a way to engineer thermally robust spin current generating alloys for application at elevated temperatures.

## Results

### High temperature ST-FMR measurements

**Figure 1:**
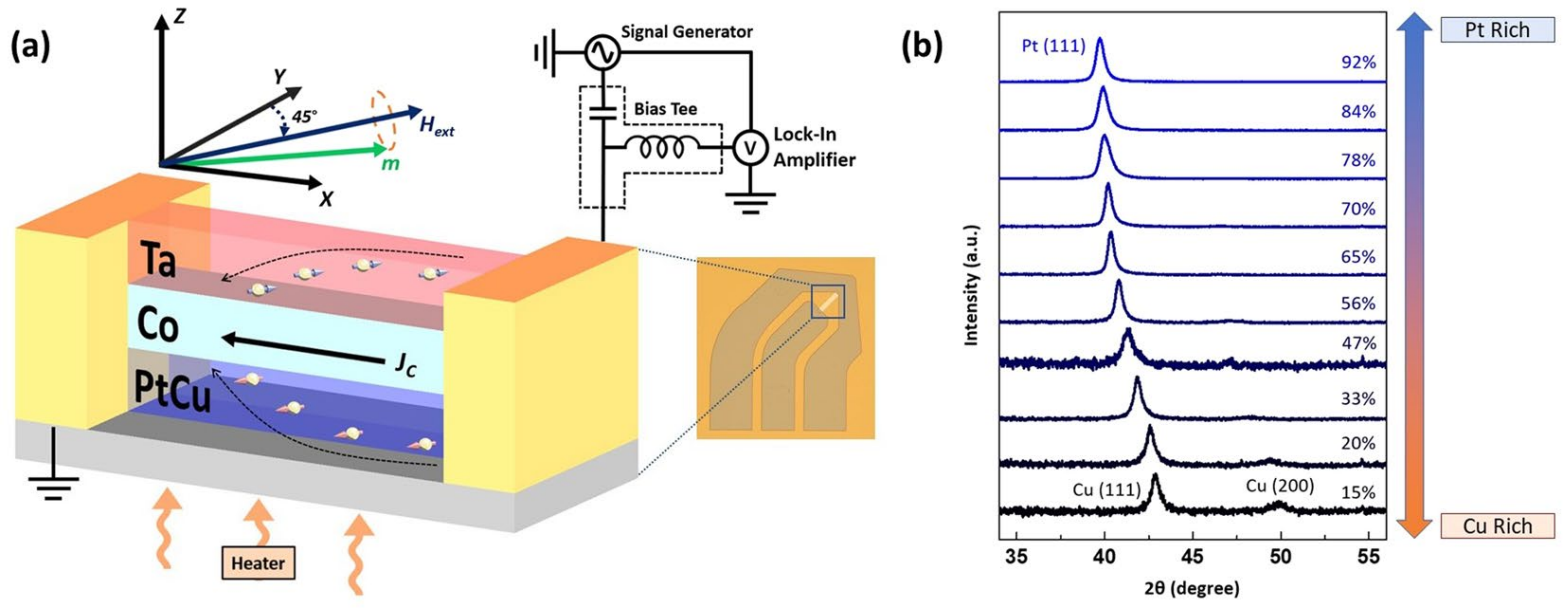
(**a**) Schematic illustration of high temperature ST-FMR setup. Optical image of the device is as shown in the inset. (**b**) X-ray diffraction pattern of 80 nm thick Pt_*x*_Cu_1−*x*_ samples showing the shift between the Pt(111) to the Cu(111) peak.

The films used in this study were deposited using magnetron sputtering onto silicon substrates at room temperature. Stack structure of substrate/Pt_*x*_Cu_1-*x*_(5 nm)/Co(5 nm)/Ta(5 nm) was fabricated for temperature investigation. Ta was introduced as the spin current source to compliment Pt_*x*_Cu_1−*x*_ due to their opposite spin Hall angle signs^4,15,21^. The Pt_*x*_Cu_1−*x*_ alloys are obtained by co-sputtering both Pt and Cu and its atomic composition is varied by adjusting their sputtering power. X-ray diffraction (XRD) results for 80 nm thick Pt_*x*_Cu_1−*x*_ films on a continuous Si substrate are shown in Fig. 1b. Similarly, in the case of pure Pt and Cu, Pt_*x*_Cu_1−*x*_ had a face-centered-cubic (fcc) structure with the Pt_*x*_Cu_1−*x*_ (111) peak gradually shifting from the Pt (111) to the Cu (111) with increasing Cu concentration. At around 70% Pt compositions, Pt_*x*_Cu_1−*x*_ (200) peak becomes observable and its intensity gradually increased with decreasing Pt_*x*_Cu_1−*x*_ (111) peak as it transited to the Cu-rich region (See Supplementary). From this, it can be concluded that the fcc (111)-textured alloy remained unchanged between the transition of Pt-rich and Cu-rich films, which indicated that the two elements mix well in their binary alloys.

**Figure 2:**
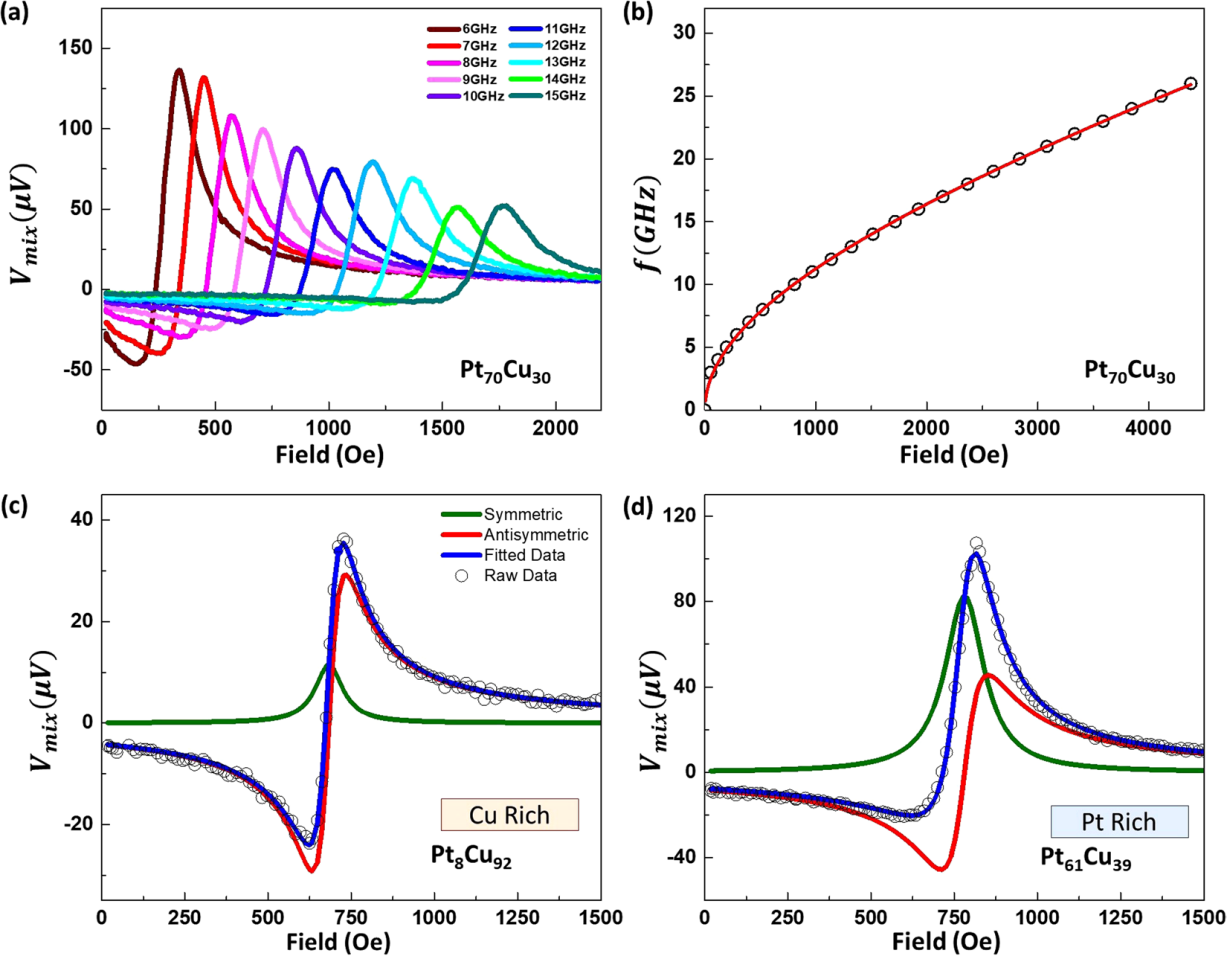
(**a**) Measured ST-FMR spectra of Pt_60_Cu_40_(5 nm)/Co(5 nm)/Ta(5 nm) trilayer device for frequency from 6 to 15 GHz with nominal input power of 18 dBm. (**b**) Fitting of Kittel equation. (**c**,**d**) ST-FMR Lorentzian fitting of ST-FMR spectra of Pt_*x*_Cu_1−*x*_ (5 nm)/Co(5 nm)/Ta(5 nm) trilayer for *x* = 8% and 61% and a microwave frequency of 9 GHz.

In this study, the spin torque ferromagnetic resonance (ST-FMR) was deployed to determine the Gilbert damping, $\alpha$, and spin Hall efficiency, ${\theta }_{eff}$_._ When the FMR condition was met, the magnetization precession driven by the torques will result in the maximum oscillation of the resistance caused by anisotropic magnetoresistance (AMR) in the Co layer. When coupled together with the oscillating current, a rectified d.c. voltage was produced and detected using a lock-in amplifier with amplitude modulation of the RF signal. Figure 1a illustrates the high temperature ST-FMR setup used with an inset showing an optical image of the device consisting of a microstrip (10 × 50 μm) tilted at 45° with its coplanar waveguide (CPW) electrode (For more information on the fabrication, see Methods). RF charge current was injected through the long axis of the stripe while an in-plane external magnetic field ${H}_{ext}$ was swept. The longitudinal RF current passing through the device generates a transverse spin current which was then injected into the adjacent Co layer. The magnetization of Co experienced two torques induced by the RF current; an in-plane torque and an out-of-plane torque^25^. The measurement was performed at a microwave power of 18 dBm and measured between 6 to 20 GHz in steps of 1 GHz. Figure 2a. shows a segment of the measured ST-FMR spectra with varying current frequencies for Pt_60_Cu_40_ (5 nm)/Co (5 nm)/Ta (5 nm).

The measured rectified d.c. voltage ${V}_{mix}$ is expressed as

1 $${V}_{mix}=S\frac{{(\Delta H/2)}^{2}}{{({H}_{{\rm{ext}}}-{H}_{{\rm{res}}})}^{2}+{(\Delta H/2)}^{2}}+A\frac{({H}_{{\rm{ext}}}-{H}_{{\rm{res}}})(\Delta H/2)}{{({H}_{{\rm{ext}}}-{H}_{{\rm{res}}})}^{2}+{(\Delta H/2)}^{2}},$$

where $\Delta H$ and ${H}_{ext}$ are the spectra width and the resonance field and *S* and *A* are magnitude the symmetric and anti-symmetric components of the ${V}_{mix}$ respectively^21^. For ST-FMR signal, the *S* component arises from the damping-like torque while the Oersted field and the field-like torque contributes to the *A* component via the following relations;

2 $$S={V}_{0}\frac{\hslash {J}_{{\rm{s}}}}{2e{\mu }_{0}{M}_{{\rm{s}}}t},$$

3 $$A={V}_{0}({H}_{{\rm{rf}}}+{H}_{{\rm{FL}}})\sqrt{1+\frac{4\pi {M}_{{\rm{eff}}}}{{H}_{{\rm{ext}}}}.}$$

where ${V}_{0}$ is a constant, ${J}_{{\rm{s}}}$ is the spin current density, ${\mu }_{0}=4\pi \times {10}^{-7}\,{{\rm{Hm}}}^{-1}$ is the magnetic permeability in free space, *t* is the thickness of the ferromagnetic (FM) layer, ${M}_{{\rm{s}}}$ is the saturation magnetization of the FM layer, ${M}_{eff}$ is the effective magnetization, ${H}_{rf}$ is the Oersted field and ${H}_{FL}$ is the effective field generated by field-like torque. Figure 2c,d are examples of the Lorentz ST-FMR fitting equation for Cu-rich and Pt-rich trilayer system respectively. The red and the green solid curves are the extracted symmetric and antisymmetric voltage contributions. The fitted curve for the summation of both contributions is fitted in blue and it overlaps well with the raw data indicating a good fit.

Fitting the measured spectra to Eq. (1), the frequency dependence of properties such as resonance field ${H}_{res}$, linewidth $\Delta H$, symmetric component *S*, and anti-symmetric component *A* was obtained. Figure 2b shows the frequency dependence of the resonance field as described by the in-plane Kittel equation^26^,

4 $$f=\frac{\gamma }{2\pi }\sqrt{({H}_{{\rm{res}}})(4\pi {M}_{{\rm{eff}}}+{H}_{{\rm{res}}})}.$$

As the composition of Pt increases indicating that there is an enhancement of the surface anisotropy constant $\,{K}_{{\rm{S}}}$ with the introduction of a heavy metal (See Supplementary). This can be attributed to a stronger spin-orbit coupling (SOC) when more Pt is present in the alloy. The FMR linewidth shows a linear dependence with the frequencies measured; indicating that the two-magnon scattering mechanism had negligible contribution in our samples^27^. Subsequently, the relation between frequency and the linewidth was examined using the following equation^28^,

5 $$\Delta H=\Delta {H}_{0}+\frac{4\pi \alpha }{\gamma }f,$$

where $\alpha$ is the effective Gilbert damping parameter and $\Delta {H}_{0}$ is the inhomogeneous broadening term originating from sample imperfections which are assumed to be frequency independent.

### Temperature dependence of Gilbert damping

**Figure 3:**
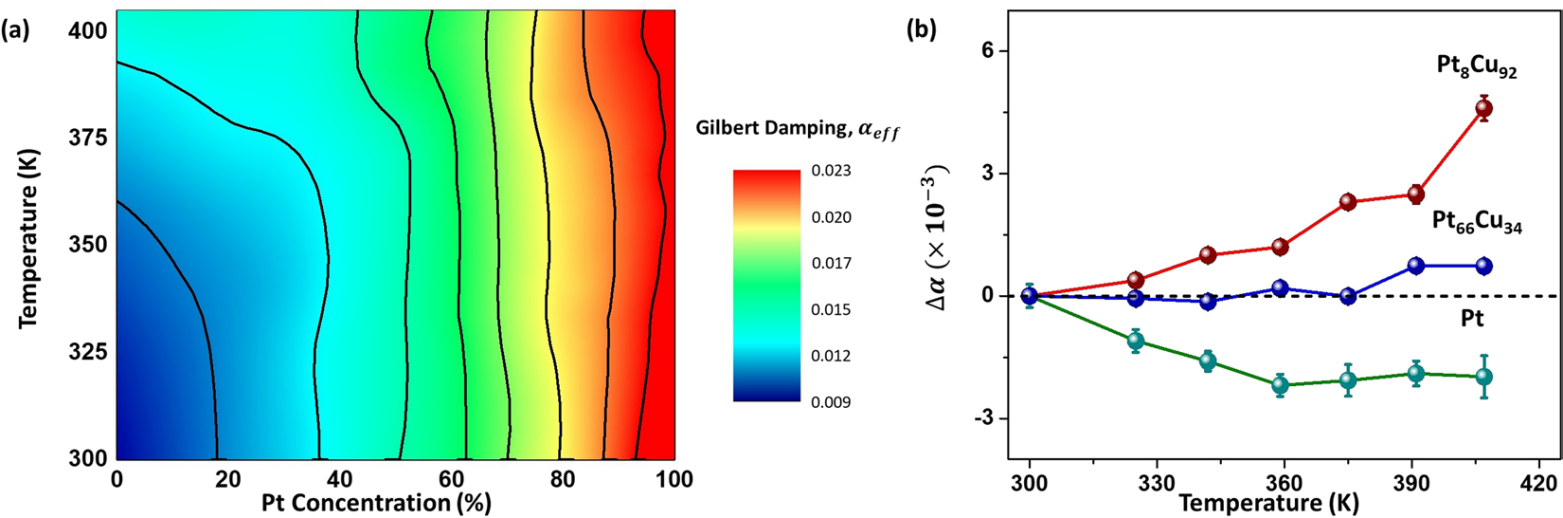
(**a**) Contour plot of $\alpha$ for Pt_*x*_Cu_1−*x*_ (5 nm)/Co(5 nm)/Ta(5 nm) trilayer device with temperature for varying Pt concentrations. (**b**) $\Delta \alpha$ of Pt_*x*_Cu_1−*x*_ (5 nm)/Co(5 nm)/Ta(5 nm) for *x* = 8%, 66% and 100%.

For device application, damping parameters should ideally be constant across the operating temperature range to maintain a consistent switching. The effective Gilbert damping constant of the trilayer system is given by $\alpha ={\alpha }_{int}+{\alpha }_{SP}$, where ${\alpha }_{int}$ is the intrinsic Gilbert damping contribution from Co and ${\alpha }_{SP}$ is the damping introduced by spin pumping effect due to the adjacent heavy metals^29,30^. Damping contribution from the proximity effect is assumed to be negligibly small as it only affects the first atomic layer of Pt^27,31–34^. From Eq. (5) using the slope of the linear relation between the linewidth and frequency, the effective Gilbert damping can be calculated. Figure 3a shows the calculated damping constants for varying Pt concentration at different temperatures. The damping constant increases with higher Pt concentration which is in good agreement with the trend observed in prior reports^15,35^. This could be due to the enhanced spin pump effect and interface anisotropy for elements with larger SOC^29,30^.

It is known that ${\alpha }_{int}$ is directly proportional whereas while ${\alpha }_{SP}$ is inversely proportional to temperature^36^. Here, $\Delta \alpha$ refers to the change in Gilbert damping constant due to an increase in temperature with respect to room temperature. Figure 3(b) shows $\Delta \alpha$ of Pt_*x*_Cu_1−*x*_ at different Pt composition. When the alloy is within the Cu-rich regime, the increase in damping due to ${\alpha }_{int}$ is much greater than the decrease from ${\alpha }_{SP}$. Therefore, this leads to an overall increase in damping $(\Delta {\alpha }_{int} > \Delta {\alpha }_{SP})$. Upon increasing the Pt concentration beyond 45%, the Pt-rich regime is established. The magnitude change for the two main damping contribution at this point are approximately the same, giving rise to near zero increment in damping with the change in temperature $(\Delta {\alpha }_{int}\approx \Delta {\alpha }_{SP})$. As Pt starts to saturate towards 100% concentration, ${\alpha }_{SP}$ begins to dominate and the overall damping decreases with temperature $(\Delta {\alpha }_{int} < \Delta {\alpha }_{SP})$.

### Temperature dependence of spin Hall efficiency

To calculate the spin Hall efficiency of the trilayer system, we implement the line-shape method using^21^

6 $${\theta }_{{\rm{eff}}}\cong \frac{S}{A}\left(\frac{e{\mu }_{0}{M}_{S}td}{\hslash }\right){\left(1+\frac{4\pi {M}_{{\rm{eff}}}}{{H}_{{\rm{res}}}}\right)}^{\frac{1}{2}}.$$

This lineshape method is the most suitable for bilayer structures with negligible field-like contribution. When used in a trilayer structure, Oersted field generated from the heavy metal layers at the top and bottom will result in cancellation and thus result in an underestimate of the ${\theta }_{eff}$^37^. To account for this, a correction factor of $1/[1+({H}_{{\rm{FL}}}/{H}_{{\rm{Oe}}})]$ can be added where ${H}_{{\rm{FL}}}$ is the fieldlike spin orbit torque and ${H}_{{\rm{Oe}}}$ is the Oersted field generated^38,39^. Because the resistivity of Ta (216 μΩ cm) is much higher than that of Pt_*x*_Cu_1−*x*_ (10–51 μΩ cm, see supplementary), the current density in Ta is small. Thus, the Oersted field attributed by Ta is ignored as ${H}_{{\rm{FL}}}/{H}_{{\rm{Oe}}}$ is negligible. With the assumption that the top 3 nm of Ta forms TaO_*x*_, the ${\theta }_{eff}$ of Pt/Co/Ta measured at room temperature in our work is 0.24 ± 0.03 which is in agreement with previous works suggesting that our approximation is appropriate^40^.

**Figure 4:**
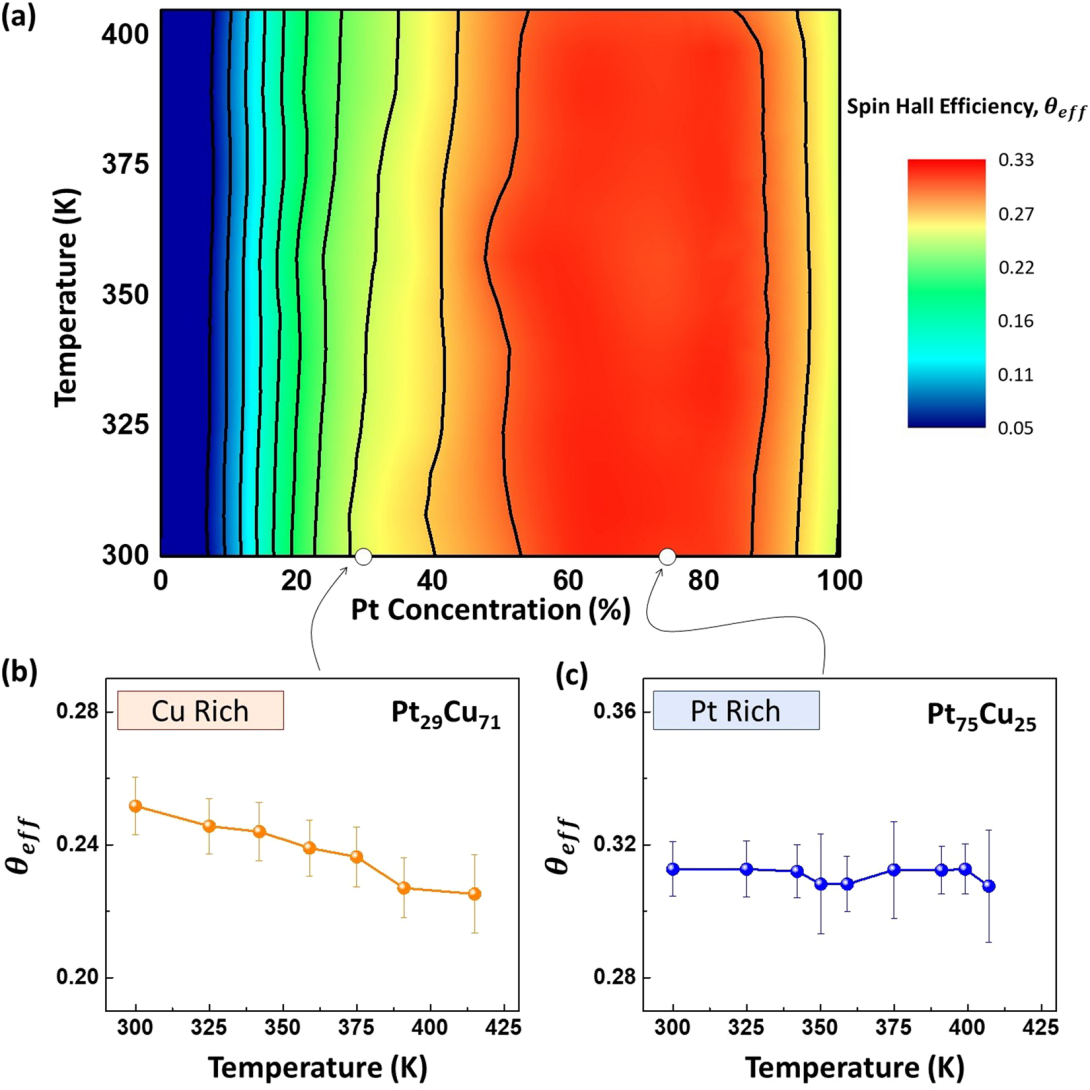
(**a**) Contour plot of ${\theta }_{eff}$ for Pt_*x*_Cu_1−*x*_ (5 nm)/Co(5 nm)/Ta(5 nm) trilayer device with temperature for varying Pt concentrations. (**b**) and (**c**) Temperature dependence of ${\theta }_{eff}$ for Cu-rich and Pt-rich Pt_*x*_Cu_1−*x*_ (5 nm)/Co(5 nm)/Ta(5 nm) trilayer device with *x* = 29% and 75% respectively.

Figure 4a summarizes the ${\theta }_{eff}$ across different Pt concentration at elevated temperatures. Here, we observe that the ${\theta }_{eff}$ has a parabolic dependence with the Pt concentration and this is attributed to the additional extrinsic SHE through alloying^15^. At room temperature, Pt_*x*_Cu_1−*x*_/Co/Ta has a peak ${\theta }_{eff}$ of 0.32 ± 0.03 at *x* = 67% (See supplementary). As shown in Fig. 4b,c, the Cu-rich alloy is more temperature sensitive and its ${\theta }_{eff}$ deteriorates more significantly with increasing temperature as compared to the Pt-rich alloy. This decrease in ${\theta }_{eff}$ is most pronounced when *x* = 15 to 40% as shown by the slightly slanted vertical lines in Fig. 4a, which is a result of the predominant side-jump scattering contribution with this region. Side-jump scattering is temperature dependent while intrinsic and skew scattering contribution are temperature independent. Skew scattering contribution is more dominant when the alloy has a dominating element within the composition^41–43^. At *x* = 75%, the extrinsic and intrinsic SHE is maximized while being temperature insensitive.

### Spin transparency and switching current density relation

**Figure 5:**
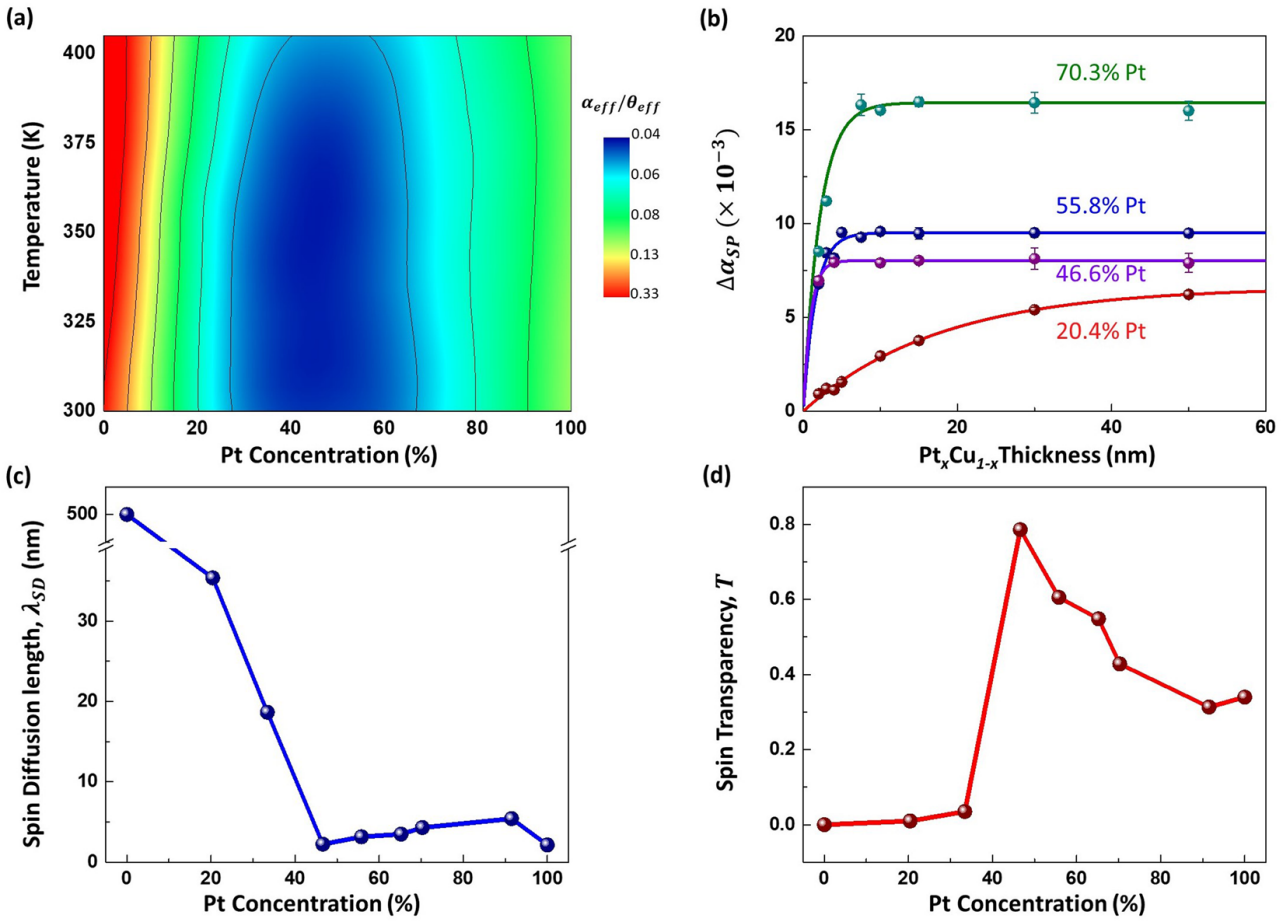
(**a**) Contour plot of ${\alpha }_{eff}/{\theta }_{eff}$ ratio for Pt_*x*_Cu_1−*x*_(5 nm)/Co(5 nm)/Ta(5 nm) trilayer device with temperature. (**b**) Damping parameter due to spin pumping of Pt_*x*_Cu_1−*x*_(*t* nm)/Co(20 nm)/Ru(5 nm) with varying *t* thickness of Co for *x* = 20%, 47%, 56% and 70%. (**c**,**d**) Alloy composition dependence of spin diffusion length and spin transparency, respectively, for Pt_*x*_Cu_1−*x*_(5 nm)/Co(20 nm)/Ru(5 nm)^44–46^.

Since the optimal spin hall efficiency and damping constant occur at different Pt concentration, there is a need to analyze and characterize them such that the optimal SOT efficiency for current-induced switching application at elevated temperature can be determined. To do so, the critical switching current density, ${J}_{c0}$, was evaluated at various temperature. The required ${J}_{c0}$ for in-plane magnetization switching using SOT is given by

7 $${J}_{c0}\approx \frac{2e}{\hslash }\frac{\alpha }{{\theta }_{eff}}\left(\frac{4\pi {M}_{eff}}{2}\right){M}_{{\rm{s}}}t,$$

which is proportional to ${\alpha }_{eff}/{\theta }_{eff}$. By plotting the ratio at different temperatures as shown in Fig. 5a, the optimal Pt concentration that has the lowest critical switching current density was found at *x* = 45%. This occurs at the transition between the Cu-rich and Pt-rich regime where the alloy becomes more thermally robust. To further understand the reason behind the optimal Pt concentration, the spin diffusion length and the spin transparency was characterized.

The thickness dependence of the heavy metal and damping can be described by^30^

8 $${\alpha }_{eff}={\alpha }_{int}+\frac{g{\mu }_{B}}{4\pi {M}_{S}({t}_{Co}-{t}_{d})}{G}_{{{\rm{Pt}}}_{x}{{\rm{Cu}}}_{1-x}}^{\uparrow \downarrow }(1-{e}^{\frac{-2{t}_{{{\rm{Pt}}}_{x}{{\rm{Cu}}}_{1-x}}}{\lambda }}),$$

where $g$ is the g-factor, ${t}_{d}$ is the magnetic dead layer thickness, ${G}_{{{\rm{Pt}}}_{x}{{\rm{Cu}}}_{1-x}}^{\uparrow \downarrow }$ is the effective spin-mixing conductance due to Pt_*x*_Cu_1−*x*_ and $\lambda$ is the spin diffusion length. Figure 5b shows the change in damping, $\Delta {\alpha }_{SP}$, of Pt_*x*_Cu_1−*x*_(*t* nm)/Co(20 nm)/Ru(5 nm) measured using FMR for varying *t* thickness of Co at different Pt concentration due to enhanced damping from alloy. Fitting the data with Eq. (8), the spin diffusion length for different Pt concentration was obtained. Figure 5c indicates that around Pt_45_Cu_55_, the spin diffusion length is the shortest at 1.9 ± 0.2 nm. The spin transparency can be calculated by the following model^46^,

9 $$T=\frac{{G}_{{{\rm{Pt}}}_{x}{{\rm{Cu}}}_{1-x}}^{\uparrow \downarrow }\,\tanh \left(\frac{d}{2\lambda }\right)}{{G}_{{{\rm{Pt}}}_{x}{{\rm{Cu}}}_{1-x}}^{\uparrow \downarrow }\,\coth \left(\frac{d}{\lambda }\right)+\frac{\sigma }{\lambda }\frac{h}{2{e}^{2}}}$$

where *T* is the spin transparency and $\sigma$ is the electrical conductivity of Pt_*x*_Cu_1−*x*_. The electrical resistivity of Pt_*x*_Cu_1−*x*_ as a function of Pt concentration has a parabolic relation that follows the Nordheim rule for homogenous solid solutions (See supplementary). In Fig. 5d, we observe that *T* peaks at 0.85 ± 0.09 when the Pt concentration is at *x* = 45%. This peak indicates that there is a relation between the critical switching current density and spin transparency. Improvement in spin transparency between the interface of the FM and the HM results in an increase in spin current propagation, thus reducing the switching current density^46^.

In summary, we have investigated the damping constant and spin Hall efficiency of Pt_*x*_Cu_1−*x*_ at elevated temperatures and discovered that they can be modulated by adjusting the temperature and alloy composition. Pt_*x*_Cu_1−*x*_ that have higher Pt content are less sensitive to temperature, but they suffer a tradeoff with higher damping. By tuning the alloy composition to Pt_47_Cu_53_, the minimum ratio of ${\alpha }_{eff}/{\theta }_{eff}$ can be achieved, which could reduce the switching current density for magnetization reversal. The temperature dependence of effective damping is also negligible due to the counteracting relationship between ${\alpha }_{int}$ and ${\alpha }_{SP}$. The spin diffusion length is at its smallest at 1.9 ± 0.2 nm and has high spin transparency of 0.85 ± 0.09. Therefore, our work here has demonstrated a method of characterizing the spin Hall channel material and its optimization for device application at elevated temperatures.

## Methods

### Sample preparation

The films were sputtered onto thermally oxidized Si substrates with (100) orientation using an Ar pressure of 2 mTorr and a base pressure <5 × 10^−8^ Torr. Two-inch-diameter targets were used. The Pt_*x*_Cu_1−*x*_ alloys were deposited by co-sputtering both Pt and Cu simultaneously and its atomic composition is varied by adjusting their sputtering power. The Pt target is varied between 20 to 100 W and Cu target is from 50 to 150 W. Energy-dispersive X-ray (EDX) spectroscopy was used to verify the percentage composition of the alloys. The samples are then patterned into 10 × 50 μm rectangular micro-strips using electron beam lithography (EBL) and Ar ion etching, thereafter coplanar waveguide (CPW) is deposited onto the stripes.

### ST-FMR characterization

RF charge current from a Keysight N5183B analog signal generator is injected into the CPW electrode through ground-signal-ground (GSG) RF probes which will flow along the long axis of the stripe. The microwave power was fixed at 18 dBm and the measured frequencies are varied between 6 to 20 GHz with an increment step size of 1 GHz. An in-plane external magnetic field ${H}_{ext}$ is swiped while injecting current at 45° relative to one another. The rectified d.c. voltage is then passed through a bias tee and into Zurich instruments lock-in amplifier for detection. Resistance of the devices was measured using a Keithley 2400 multimeter. For high temperature ST-FMR measurements, the non-magnetic heating element is positioned directly below the sample stage preheating the sample for 20 minutes before and throughout the measurement such that equilibrium temperature is achieved.

## Supplementary information

Supplementary Information.
