# Supplementary material for: Thermal behavior of spin-current generation in PtxCu1-x devices characterized through spin-torque ferromagnetic resonance
Source: Sci Rep. 2020 Jun 15;10:9631. doi: 10.1038/s41598-020-66762-8 (PMC7295739; doi:10.1038/s41598-020-66762-8)
Supplement: Supplementary file 1 — Supplementary Information. [file 41598_2020_66762_MOESM1_ESM.docx]

Supplementary Material

**Thermal behavior of spin-current generation in Pt*_x_*Cu_1-_*_x_* devices characterized through spin-torque ferromagnetic resonance**

G.D.H. Wong^1,2^, W.C. Law^1,2^, F.N. Tan^1,2^, W.L. Gan^1^, C.C.I. Ang^1^, Z. Xu^1^,

C.S. Seet^2^, W.S. Lew^1*^

*^1^School of Physical & Mathematical Sciences, Nanyang Technological University,*

*21 Nanyang Link, Singapore 637371*

*^2^GLOBALFOUNDRIES Singapore Pte, Ltd., Singapore 738406, Singapore*


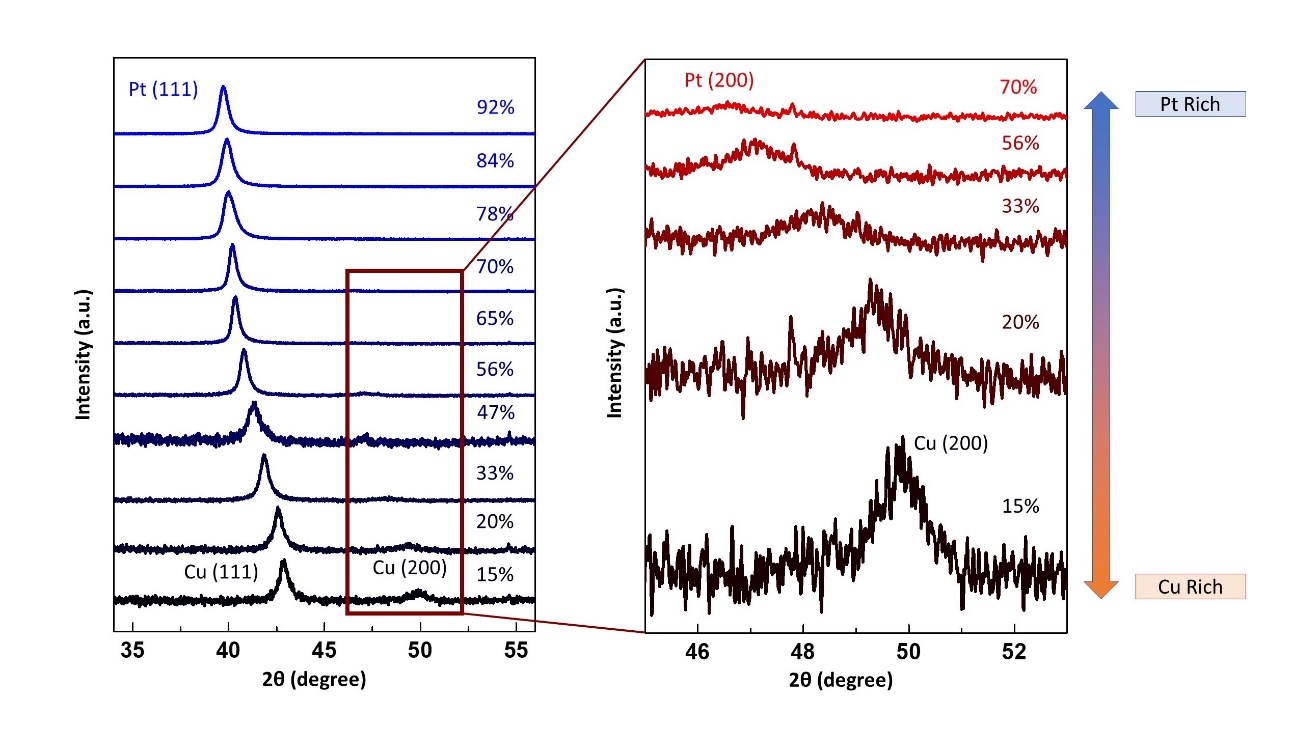
**S1. Additional Pt*_x_*Cu*_1-x_* (200) peak**

**Figure S1.** X-ray diffraction pattern of 80nm thick Pt_X_Cu_1-X_ samples showing the shift between the Pt(111) to the Cu(111) peak. Inset: Magnified region of the spectrum showing the appearing Pt_X_Cu_1-X_ (200) peak as the composition of Cu increases.

Apart from the shift in Pt*_x_*Cu*_1-x_*(111) textured peak, there exists an additional Pt_X_Cu_1-X_ (200) textured peak as shown in Fig. S1. This becomes more apparent as the alloy moves towards the Cu rich regime. The transition between the peak shifts occur gradually indicating that the alloying between the two elements are well mixed in their binary alloy.

**
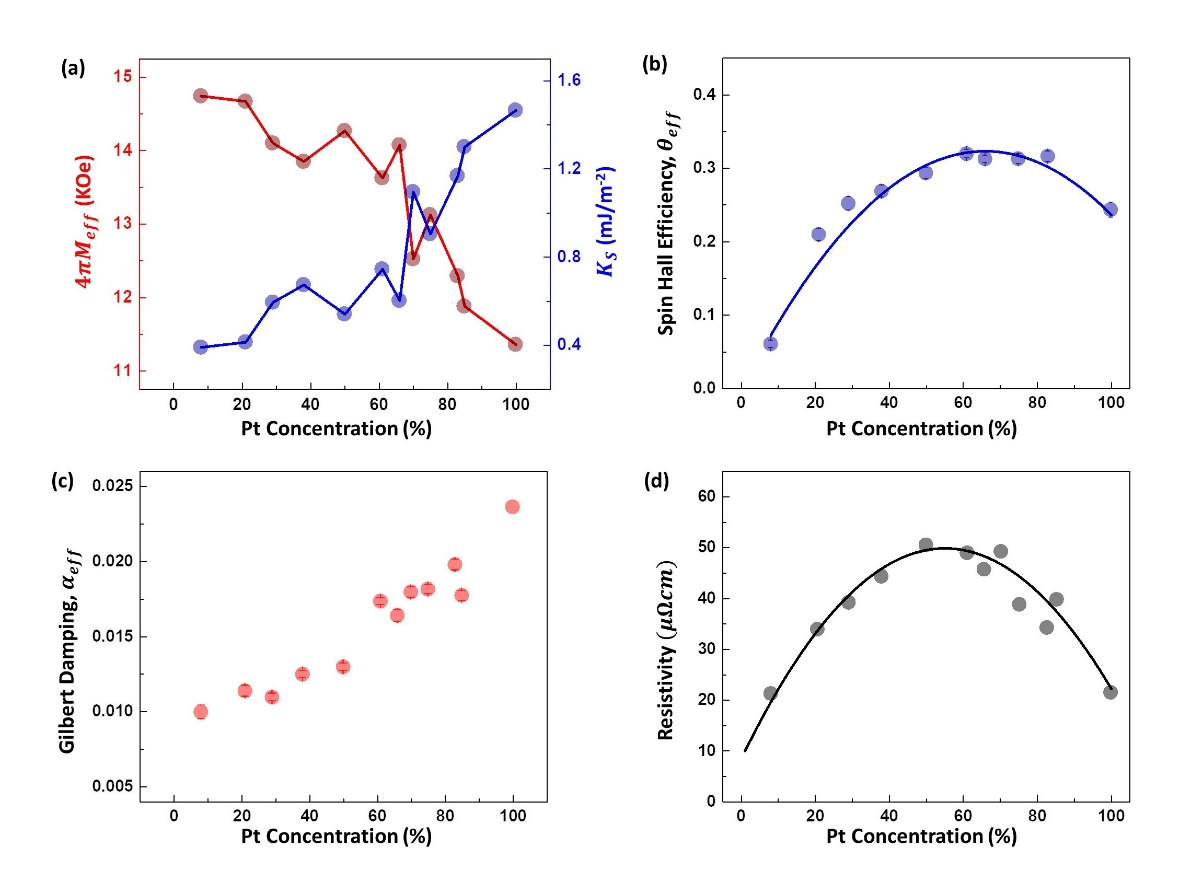
S2. ST-FMR characterization of Pt*_x_*Cu*_1-x_*(5)/Co( 5)/Ta(5) at room temperature**

**Figure S2.** (**a**) The effective magnetization $4\pi M_{eff}$ (red circles) and surface magnetic anisotropy constant $K_{\text{s}}$ (blue circles) dependence on Pt concentration in Pt*_x_*Cu_1-_*_x_*(5 nm)/Co(5 nm)/Ta(5 nm) films. (**b**) $\theta_{eff}$ at different Pt concentration extracted using the “lineshape” method ($V_{S}/V_{A}$ ratio). (**c**) $\alpha_{eff}$ at different Pt concentration calculated from the ST-FMR linewidth. (**d**) $\rho_{Pt_{x}Cu_{1-x}}$ at different Pt concentration fitted with the Nordheim relation.

The trends of the ST-FMR measurements which are performed at room temperature are shown in Fig. S2 which concurs with data from previous works^1,2^. In Figure S2a, the effective magnetization given by $4\pi M_{eff}=4\pi M_{S}-2K_{S}/M_{S}t$ decreases as Pt concentration increases while the surface anisotropy constant $K_{S}$ increases. From Figure S2b the $\theta_{eff}$ of Pt_X_Cu_1-X_ behaves in a parabolic manner as it increases upon the introduction of Cu into the alloy. This resulted from additional extrinsic SHE from skew and side jump scattering. Furthermore, as the decrease in intrinsic SHE outweighs the increase, the $\theta_{eff}$ starts to deteriorate. The Gilbert damping in Pt*_x_*Cu*_1-x_* increases as Pt concentration increases as shown in Fig. S2c. This enhanced damping is a result of spin pumping from the adjacent metal of high spin-orbit coupling. Figure S2d shows the resistivity of Pt*_x_*Cu*_1-x_* at different Pt concentration fitted to the Nordheim relation for homogenous solid;$\rho_{Pt_{x}Cu_{1-x}}=C_{1}x\left(100-x\right)+\left(\rho_{Pt}/100\right)x+\left(\rho_{Cu}/100-x\right)$, where $C_{1}$ is coefficient of parabolic relation, $\rho_{Pt}$ and $\rho_{Cu}$ are the resistivity of Pt and Cu respectively^3,4^. The data fits well with the parabolic relation which indicates that the Pt*_x_*Cu*_1-x_* alloys are homogenous.


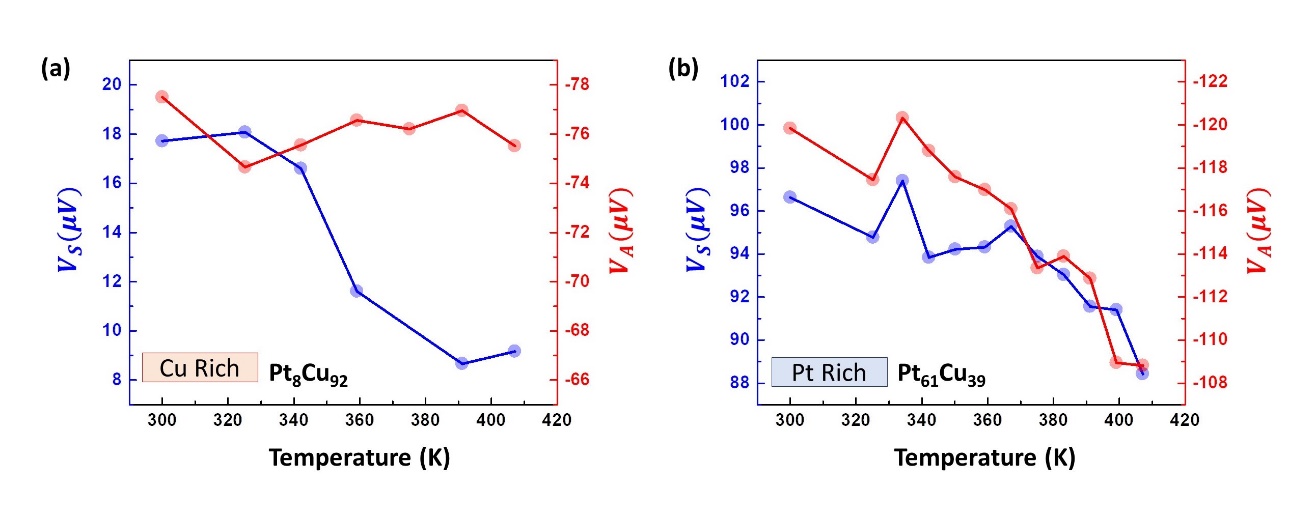
**S3. Temperature dependence of symmetric and asymmetric component**

**Figure S3.** $V_{S}$ and $V_{A}$ component extracted from the ST-FMR “lineshape” method for alloy composition of (**a**). Pt_8_Cu_92_ within the Cu rich regime and of (**b**). Pt_61_Cu_39_ within the Pt rich regime.

From Figure S3a and b, we observe that the $V_{A}$ in both the Cu-rich regime and Pt-rich regime behaves differently. When Cu is dominant, $V_{A}$ remains constant with an increase in temperature as compared to the Pt-rich regime. Since the $\theta_{eff}$ is proportional to the ratio between $V_{S}$ and $V_{A}$, $\theta_{eff}$ would have a greater impact due to elevated temperature in the case of Cu-rich alloys.

**References**

1 Ramaswamy, R. *et al.* Extrinsic spin Hall effect in Cu 1− x Pt x. *Physical Review Applied* **8**, 024034 (2017).

2 Bouloussa, H. *et al.* Pt concentration dependence of the interfacial Dzyaloshinskii–Moriya interaction, the Gilbert damping parameter and the magnetic anisotropy in Py/Cu1− xPtx systems. *Journal of Physics D: Applied Physics* **52**, 055001 (2018).

3 Nordheim, L. Zur Elektronentheorie der Metalle. II. *Annalen der Physik* **401**, 641-678, doi:10.1002/andp.19314010602 (1931).

4 Chopra, K. L., Thakoor, A. P., Barthwal, S. K. & Nath, P. Electron transport properties of amorphous Cu-Ag films. *physica status solidi (a)* **40**, 247-255, doi:10.1002/pssa.2210400132 (1977).
